# Pulmonary Cavern Opening above the Clavicle

**Published:** 1831

**Authors:** 


### PULMONARY CAVERN OPENING ABOVE THE CLAVICLE.

[Hôpital St. Louis.]

It is not long since we laid before our readers some particulars of a case, where a pulmonary cavern communicated with the external air through an opening between two ribs, and through which aperture the patient (Mr. Macklin) expectorated (by coughing and breathing) large quantities of purulent and fetid matters for many weeks before his death.

The following case bears some analogy to the one we put on record.

Francis Derouin, aged 18 years, entered the St. Louis on the 24th Jan. 1831. He stated that he had often laboured under catarrhal affections in Winter time. The present complaint had commenced, eighteen months previously, with pain in his back and under the shoulder-blade of the side affected. In the course of five or six months a tumour appeared on the left side of the neck, for which the patient entered the HÔTEL DIEU in September 1830, where he remained three months under M. Breschet, who punctured the tumour and evacuated its purulent contents. He remained in the HÔTEL DIEU about three months, and then repaired to the Hôpital St. Louis. On examination there, they found a fistulous opening above the left clavicle, near the insertion of the sterno-cleido muscle. During the examination the patient coughed two or three times, and, in each of these efforts, threw out a quantity of purulent matter, mixed with some bubbles of air. On further examination, the patient presented all the essential characters of phthisis. A probe passed readily to a depth of four inches, without causing any pain. After some time the pus became very fetid, and the patient's breath partook of the same. The force of the air passing out of the cavern also increased, so that, by the 20th May, he could blow out a candle through the fistula. The inspiration was always per vias naturales. He died exhausted on the 6th of June.

*On dissection,* several of the ribs were found to be carious, and an enormous cavity was discovered, filled with pus, and lined with a secreting membrane, and communicating with some bronchial tubes. Tubercles were also found of different sizes in both lungs.—*Rev. Med.*

---

LXVI

